# Acknowledgement to Reviewers of *Biomimetics* in 2019

**DOI:** 10.3390/biomimetics5010006

**Published:** 2020-02-07

**Authors:** Biomimetics Editorial Office

**Affiliations:** MDPI AG, St. Alban-Anlage 66, 4052 Basel, Switzerland; biomimetics@mdpi.com

The editorial team greatly appreciates the reviewers who have dedicated their considerable time and expertise to the journal’s rigorous editorial process over the past 12 months, regardless of whether the papers are finally published or not. In 2019, a total of 74 papers were published in the journal, with a median time to first decision of 31.5 days and a median time from submission to publication of 72.5 days. The editors would like to express their sincere gratitude to the following reviewers for their generous contribution in 2019:
Adamatzky, AndrewLee, Bruce P.Akbarzadeh, MasoudLi, QiaochuAlmeida, HenriqueLiauzun, CedricAlves, FabioLieberzeit, Peter A.Ameloot, MarcelLiu, Cheng-YangArena, MaurizioLiu, HaijunAsano, YukiLongden, KitAshley, JonLosada-Perez, PatriciaAtrián-Blasco, ElenaMalachowski, JerzyAyres, PhilMankin, Richard W.Badarnah, LidiaMaroto-Gómez, MarcosBaena, Francisco ManuelMarquardt, DrewBahri, MitraMarrazzo, PasqualeBajpai, AnkurMcHenry, MatthewBanaś, MichałMechrez, GuyBarnea, OferMeghdari, AliBax, Noortje A. M.Meldal, MortenBecerra Permuy, Jose AntonioMendelson, LeahBelitsky, Jason M.Michaud, VeroniqueBellardita, CarmeloMiller, LauraBenaouicha, MustaphaMishina, YujiBenetatos, PanayotisMoosavian, AminBenvenuti, DavideMoutsopoulou, AmaliaBerns, KarstenMyers, Damian E.Beysens, DanielNelson-Cheeseman, BrittanyBhasin, DeveshNicolas, CyrilBhounsule, Pranav A.Niiyama, RyumaBirkedal, HenrikNiu, RanBogach, NataliaOh, Dongyeop X.Bortot, ElianaOstasevicius, VytautasBotez, R. M.Ozkan-Aydin, YaseminButenkov, Sergey A.Pellerin, ChristianCanfield, StephenPetrović, MilicaCaramella, CarlaPettit, ThomasCarl, TimoPotemkin, D. I.Carrasco, SergioPrilutsky, BorisChayaamor-Heil, NatashaQian, Jin-yuanChen, BilinQiaorui, SiCheng, I-FangRegan, FionaCheung, BarryRezaeiha, AbdolrahimCho, NamjoonRibeiro, Adriana SantosClark, Christopher J.Rossi, AndreaCompain, PhilippeRoy Chowdhury, SandipanConti, DanielaŠafarič, RikoCvrček, LadislavSain, SunandaDe Gaspari, AlessandroSamoylova, Tatiana I.De Stefano, LucaSantra, SwadeshmukulDeLaurier, AprilSarbu, AndreiDelbianco, MartinaSarna, TadeuszDeravi, LeilaSauret, EmilieDoctor, KhoshravSchuster, BernhardDuchi, SerenaSek, SlawomirElahi, HassanSeriani, StefanoEllison, MichaelShamonin, MikhailEngelbrecht, AndriesShamshirband, ShErgin, F. GökhanShibahara, MakotoFaiña, AndresShu, QingyanFarinola, GianlucaSilberstein, MeredtihFazal, ZeeshanSiniscalchi, Sabato MarcoFeldman, DaleSitti, MetinFernandes, Carlos M.Socha, JakeFlammang, BrookeSong, JuhaFolio, DavidSprecher, AaronGallo, MauroSprynskyy, MyroslavGavazzo, PaolaStepanov, Gennady V.Gavioli, LucaStokke, Bjørn TorgerGräbner, DanielStrube, OliverGradov, Oleg V.Su, Tsung-chowGreen, MelissaSullivan, TimothyGrobman, YashaSuzuki, AussieHabib, MakiTakagi, DaisukeHammond, Frank L.Takuma, TakashiHanaor, Dorian A. H.Tangorra, James LouisHanke, WolfTesar, VaclavHariri, Hassan HusseinTsuboko, YusukeHempel, UteTurco, AntonioHes, DominiqueValiev, A. R.Hess, DennisVan Caam, ArjanHigginson, Cody JamesVijayavenkataraman, SanjairajHilborn, JönsVincent, JulianHolten-Andersen, NielsVukomanovič, MariijaHoover, AlexWada, TohruHumphries, StuartWaite, J. HerbertIjspeert, AukeWang, QimingIop, LauraWang, HongjunIsaev, AbdulgalimWang, XiangIshikawa, TakujiWarburton, NatalieJaecques, SiegfriedWebb, BarbaraJi, Ai-hongWei, QiJurman, GiuseppeWeihs, DanielKadapa, ChennakesavaWilliams, JohnKakugo, AkiraWu, JieKapadia, ApoorvaWysokowski, MarcinKasem, HaytamYakout, MostafaKhan, Muhammad AhmedYamanobe, NatsukiKim, Hwa SooYang, Yi-YuanKim, DonghyunYousefi, KianooshKlimek, KatarzynaZari, MaibrittKomarova, SvetlanaZboinska, Malgorzata A.Körner, AxelZhang, JiayingKouziokas, Georgios N.Zhang, HaochunKrólczyk, GrzegorzZhang, YanLamas, María IsabelZhu, KuiLatif, TahmidZuppinger, ChristianLee, Hyoungsoon


